# A National Health Insurance Program for the United States

**DOI:** 10.1371/journal.pmed.0010039

**Published:** 2004-11-30

**Authors:** Don R McCanne

## Abstract

The US will spend $1.79 trillion on health care in 2004, yet 44 million Americans remain uninsured. What the country needs, argues McCanne, is publicly funded universal health coverage

## Teaser

The country must abandon its fragmented system

The total projected spending on health care in the United States for 2004 is $1.79 trillion—15.5% of its gross domestic product [1]. That amounts to $6,167 per person, almost twice what most nations with comprehensive systems spend on care. Most policy analysts agree that this level of spending should be more than enough to provide all Americans with high quality, comprehensive health care. Yet the United States falls far short of these goals. What are the flaws in the United States health system that prevent Americans from receiving value from this huge health care investment? And what are the options for improvement?

## Physicians for a National Health Program

First, I should reveal my personal bias. Physicians should be well represented in the forefront of reform. As we look back on the past half century of failed health policy decisions, we see that the dominant physicians' organization in the United States, the American Medical Association (AMA), has opposed most reform measures that would result in an equitable, affordable system for everyone. Instead, the AMA has supported an agenda that promotes physicians' freedom to maximize their personal financial reward, even though those policies may deprive tens of millions of Americans access to affordable care. The AMA agenda has contributed significantly to the current high costs of American health care and to our failure to adequately address the mediocrity that characterizes health care in the United States.

Many American physicians—including myself—believe that the funding infrastructure should be redesigned to maximize heath care resource allocation for the primary benefit of patients. Because of the failure of organized medicine to advocate on behalf of our patients, we decided that a new organization was needed. We established Physicians for a National Health Program (www.pnhp.org) [2,3].

## The Uninsured and the Poorly Insured

**Figure 1:**
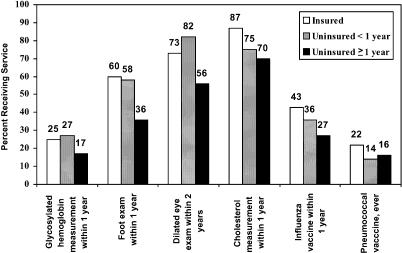
Diabetes Management among Insured and Uninsured Adults, Ages 18–64 The figure is based on data from a United States national survey of 105,764 adults in 1997 and 117,364 in 1998 [5]. The proportions have been adjusted to the demographic characteristics of the study cohort, controlling for age, sex, race/ethnicity, region, employment status, education, and income. (Reprinted, with permission, from [4], courtesy of the National Academies Press, Washington, DC, United States.)

There are 45 million Americans with no health care coverage, and not surprisingly, lack of insurance is associated with worse health outcomes [4]. About 18,000 young adults die each year because they lack health insurance [4]. The uninsured are less likely than the insured to receive the professionally recommended standard of care for their chronic diseases, such as diabetes (Figure 1) [5]. And if you have a serious health crisis while you are uninsured, you risk major debt or bankruptcy.

Even the insured are inadequately covered. Employers and individuals who purchase coverage are rebelling at the high price of insurance premiums. To maintain competitive premiums, insurers are designing products that reduce the benefits they pay out by increasing the out-of-pocket portion that patients are required to pay for services received. Insured patients may have to pay cash for care until a designated amount is reached (the deductible)—which could be thousands of dollars. In addition, patients are often required to pay a dollar amount (co-payment) or a percentage of the charges (coinsurance) each time services are received.

Insurers may also exclude specified services from coverage, such as maternity benefits or mental health services. Most insurance plans now use lists of contracted physicians and hospitals, and impose severe financial penalties for using health care providers that are not contracted. All of these measures reduce the value of insurance by shifting costs from the insurers to the patients who actually need care.

Inadequate insurance coverage is making average-income Americans poorer. A recent study found that for 29% of individuals who had average or greater-than-average incomes and were continually insured, medical bills had caused significant financial problems [6]. For those who were not continuously insured, the percentages were even higher. These financial barriers are impairing access to beneficial services. The United States insurance market is now dominated by insurance plans that provide neither adequate health security nor financial security.

## Does Higher Health Spending Mean Better Quality?

There is a widespread belief that the high spending in the United States means that high quality care is being delivered to the majority, who can afford both comprehensive coverage and the attendant out-of-pocket expenses. But international comparisons of industrialized nations have shown that the United States is in the bottom quartile of population health indicators such as life expectancy and infant mortality [7]. And in regional comparisons within the United States, increased levels of spending have not produced a commensurate improvement in health care outcomes. In fact, a recent study found that in a state-by-state comparison, there is an inverse relationship between spending and quality outcomes—the more expenditure, the worse the quality [8].

In 2000, the World Health Organization rated the United States first in its health expenditures per capita, but 37th in its overall health system performance, below most industrialized nations [9]. The United States is clearly not receiving adequate value for its health care investment.

Some contend that the poor performance of the United States system is due to the funding of health care in the private sector, and that all would be well if the government would just take over funding. But it is not quite that simple. The greater part of health care in the United States—59%—is already funded by the tax system. On a per capita basis, the public, taxpayer-funded health care expenditures alone total more than the health care spending of every other nation's public and private funding combined (with the exception of Switzerland, in which total spending per capita equals our public spending alone) [10].

## Flaws in Funding and Allocation

How can the United States spend as much as it does and end up with such mediocre health care? Of the many reasons that exist, two are particularly important. The United States has a highly flawed system of funding health care and a flawed system of allocating its health care resources.

In the United States, a multitude of private health plans cover the lucrative sector of society—low cost, healthy workers and their healthy families. But public programs must cover the higher costs of the elderly, individuals with permanent disabilities, and some low-income individuals. Since the uninsured are frequently unable to pay for the care they receive, the costs for their care are shifted to government programs or private plans, or to the charity of providers, even if unintended. The costly administrative excesses of private health plans, especially when contrasted to government programs, have been well documented [11]. This fragmented system of funding care places an even greater administrative and financial burden on the providers of health care. Although the exact amount is disputed, most policy analysts agree that replacing this fragmented system of funding care with a single, universal, publicly administered insurance program could recover 200 billion dollars or more, which are currently being wasted on useless and sometimes detrimental administrative services [11].

And what is wrong with the way that the United States allocates its resources? Many studies have confirmed that supporting a strong primary care base provides better outcomes at a lower cost [12]. But in the United States, specialized, high-technology care is heavily marketed, and providers of that high-tech care are rewarded more generously than primary care professionals. Yet studies show that these greater expenditures result in no additional benefit—and sometimes even in worse outcomes [8,13]. Excessive resources are allocated to inappropriate expansion of high-tech facilities and to training an excessive number of specialists to provide high-tech services [8,13].

## Health Care Reform

What has been the response to these deficiencies in the United States health care system? In the 1990s, the Clinton administration attempted to introduce a comprehensive system of funding universal health care. The system would have used marketplace principles in a program of managed competition, but their complicated idea pleased no one, and it was never even brought to a vote. Because of this miserable political failure, policymakers decided that any comprehensive approach should be avoided, and that reform must take place in incremental steps. To date, with the notable exception of the State Children's Health Insurance Program (http://www.cms.hhs.gov/schip), the accomplishments of these incremental health reform measures have been unimpressive.

Over the past decade, those interested in reform have been preoccupied with managed care measures and, more recently, with consumer-directed measures that increase costs to patients by requiring greater out-of-pocket spending. But these measures are designed more to control costs than to increase coverage and access. In the debate on universal coverage, three general concepts have been put forward: (1) the expansion of our current system of public and private programs, (2) the establishment of a national health service with government ownership of the system, or (3) the replacement of all current funding with a single, publicly administered, publicly funded program of social insurance that does not alter the existing ownership status of the delivery system.

The greatest political support today is for incremental expansions of our current programs, which, theoretically, would eventually result in universal coverage. There are innumerable variations of this approach. Most would increase the affordability of insurance premiums for private group and individual plans by providing financial assistance through tax policies and by modifying the benefits and coverage of the plans. Some policy analysts recommend that employers be mandated to offer coverage to their employees. Others recommend that individuals be required to purchase their own coverage. Since some individuals would be left without coverage, a public program, such as the existing Medicaid program for low-income individuals, would be used to cover everyone else. Many simulation studies have shown that these approaches could be effective in covering almost everyone, but they are the most expensive models of reform since they leave in place the administrative excesses of the fragmented system of funding care [14]. Also, to keep premiums affordable, these approaches may fall short on comprehensiveness of coverage and on the affordability of the out-of-pocket component, especially for those individuals with greater health care needs.

In contrast, simulation of both the national health service and public social insurance models of reform have shown that they would provide truly comprehensive benefits for everyone, and that they are the least expensive models [14]. By integrating funding with the health care delivery system, both models are well suited for the introduction of an integrated information technology system. Such a system would provide invaluable data to assist with decisions on resource allocation, enabling incentives to be established that would strengthen the primary care base. It would also improve capacity planning for high-tech and specialized services, thereby ensuring appropriate access without excessive queues [15].[Fig pmed-0010039-g001]

**Figure pmed-0010039-g001:**
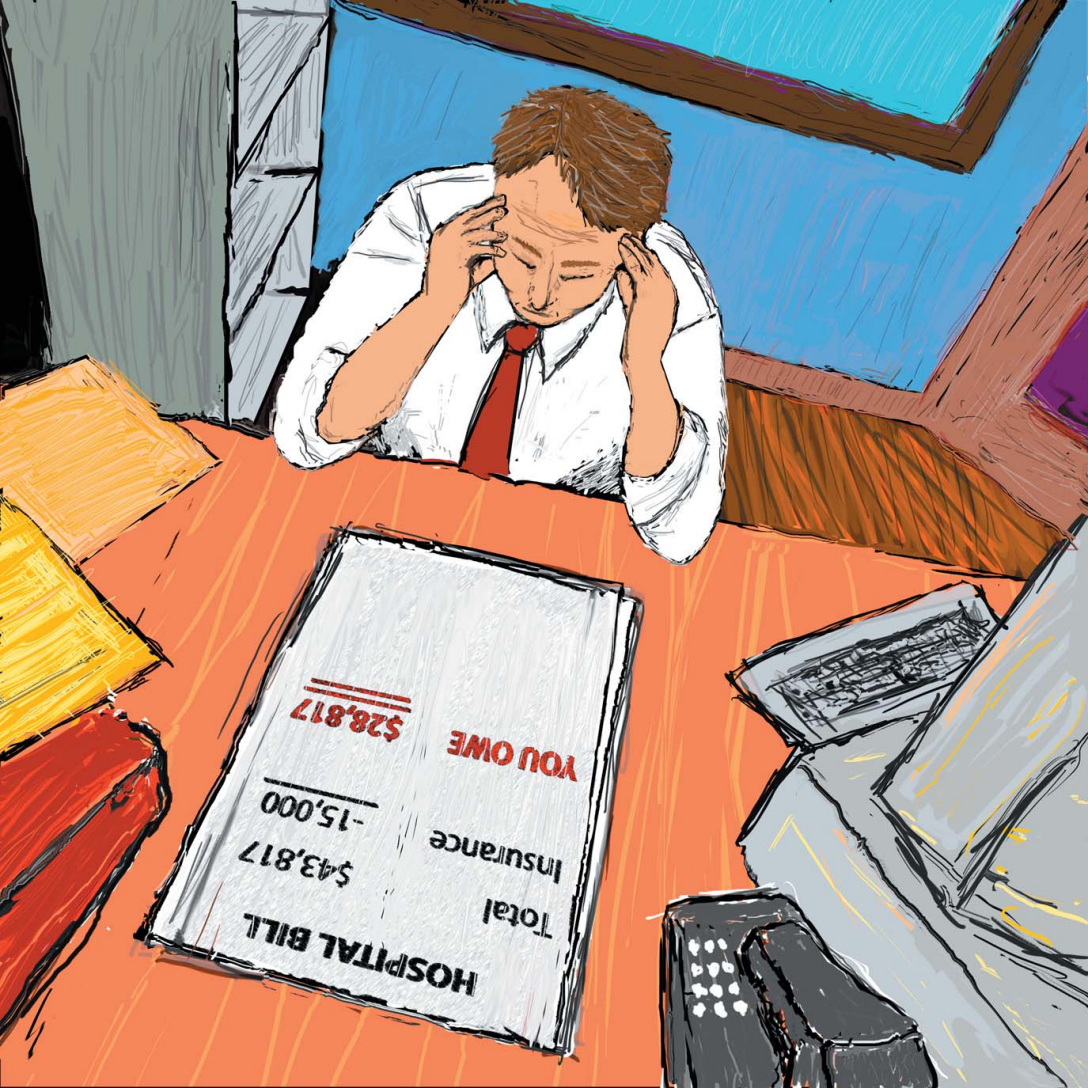
Even with insurance, a serious health crisis can lead to major debt or bankruptcy (Illustration: Rusty Howson, sososo design)

The political threshold for adopting a government-owned health service model in the United States is very high, since most citizens fear the specter of “socialized medicine.” In contrast, the Medicare program, an insurance program for the retired and for those with long-term disabilities, is very popular. There is an increasing public perception that we may need to accept a greater government role in health insurance if we are to adequately address the deteriorating status of our health care system. Correcting the flaws in Medicare and then using the program to cover everyone may be a concept that can gain political traction in the United States.

## Conclusion

Our political process is currently dominated by those who are enticed by the siren song of the market theorists and turn a deaf ear to the health policy scientists who plead for health care justice. The debate needs to focus on defining the best role for government in ensuring that people receive the best health care value. That debate needs to be guided by a thorough understanding and diligent application of sound health policy science.

The continuing deterioration of affordability, coverage, and quality in health care makes it imperative that United States policymakers broaden their reform efforts beyond the ineffectual tinkering of incrementalism. A universal, single-payer, publicly funded and publicly administered program of social insurance would ensure access to affordable, comprehensive, high-quality health care for all. It should be the standard by which any other proposals are judged. If a better proposal can be crafted, now is the time to do it. People are dying while we delay.

## Abbreviation

(AMA): American Medical Association
